# Supporting older people through Hospital at Home care: a systematic review of patient, carer and healthcare professionals’ perspectives

**DOI:** 10.1093/ageing/afaf033

**Published:** 2025-02-23

**Authors:** Anita Wong, Claudia Cooper, Catherine J Evans, Mark James Rawle, Kate Walters, Simon Paul Conroy, Nathan Davies

**Affiliations:** UCL Medical School, University College London, London, UK; Wolfson Institute of Population Health—Centre for Psychiatry and Mental Health, Queen Mary University of London, Yvonne Carter Building, Turner Street, London EC1, London E1 2AB, UK; Cicely Saunders Institute, Department of Palliative Care, Policy and Rehabilitation, King’s College London, Bessemer Road, London SE5 9PJ, UK; Sussex Community NHS Trust—Palliative Care, Brighton General Hospital, Elm Grove, Brighton BN2 3EW, UK; Academic Centre for Healthy Ageing (ACHA), Whipps Cross University Hospital, Barts Health NHS Trust, Whipps Cross Road, London E11 1NR, UK; MRC Unit for Lifelong Health and Ageing, University College London, 5th Floor, 1-19 Torrington Place, London WC1E 7HB, UK; UCL—Research Department of Primary Care and Population Health, Royal Free Hospital, London NW3 2PF, UK; MRC Unit for Lifelong Health and Ageing, University College London, 5th Floor, 1-19 Torrington Place, London WC1E 7HB, UK; St Pancras Rehabilitation Unit, Central and North West London NHS Foundation Trust—St Pancras Hospital, South Wing 4 St Pancras Way, London NW1 0PE, UK; Wolfson Institute of Population Health—Centre for Psychiatry and Mental Health, Queen Mary University of London, Yvonne Carter Building, Turner Street, London EC1, London E1 2AB, UK

**Keywords:** carers, frailty, virtual wards, hospital at home

## Abstract

**Introduction:**

Hospital at Home provides hospital-level type care at home, both remote and face-to-face by a multidisciplinary team of healthcare professionals. In practice, various different models are employed, but we do not know what older people, their family carers (carers) and healthcare professionals think of what works best for them. This review aimed to describe the various Hospital at Home models and synthesise literature exploring patient, carer and staff perspectives of Hospital at Home care for older people.

**Methods and analysis:**

A systematic review of UK studies. Medline, Embase and CINAHL and grey literature were searched from 1991 to 2024, using predetermined inclusion and exclusion criteria; data were extracted from included papers. Tabulation, thematic grouping and concept mapping of themes were used to narratively synthesise the literature.

**Results:**

Twenty studies met eligibility. Hospital at Home models included admission avoidance and early discharge. Studies were largely positive regarding Hospital at Home, with benefits including home familiarity, enabling person-centred care and shared decision-making and provision of family carer support. Challenges included staff accessibility, patient and carer anxieties regarding the safety of virtual wards, coordination across sectors and older people using technology.

**Conclusion:**

Provision of holistic, accessible and continuous care for older people in Hospital at Home services facilitated patient and carer empowerment, dignity and autonomy. There are gaps in our understanding and evidence surrounding paid care workers and informal carers’ perspectives in UK settings, especially within rigorous Hospital at Home literature.

## Key Points

- Care provision in a familiar home environment with shared decision-making was valued by all stakeholders.
- Older people, family carers and healthcare professionals’ experiences should be integrated in future research and evaluation.
- Older people and their family carers reported mainly positive experiences with hospital at home.
- Hospital at home were perceived by older people and family carers as a positive driver for patient independence and recovery.
- Research potential for developing evidence-based support for family carers in hospital at home to alleviate carer burden.

## Introduction

Better public health, medical advances and improved living conditions have led to longer lifespans [1]. In recent years, reports illustrate notable increases in potentially avoidable older adult hospitalisation and emergency admission, prolonged recovery time and inpatient stays, leading to worsening health outcomes such as delirium [2–4]. Older adults with frailty describe negative experiences in emergency care, with their needs not being met, potentially contributing to their reluctance to attend hospital in the first place [5]. This is perhaps in part due to the variable provision of gold-standard evidence-based care (comprehensive geriatric assessment) in acute hospital settings [6, 7].

The COVID-19 pandemic accelerated nationwide incorporation of teleconsultations—key to NHS England’s national Virtual Ward (VW) expansion programme which launched in 2022 [8–11]. NHS England defines VWs as technology-enabled, safe alternatives to hospital care, led by an appropriate clinical lead and a multidisciplinary team (MDT) [12, 13]. NHS’ shift of terminology from ‘Hospital at Home’ (HaH) to ‘Virtual Ward’ aimed to highlight the integration of digital technology [12, 13]. However, the Hospital at Home Society, British Geriatrics Society (BGS) and Royal College of Physicians have recently advocated for reverting back to using HaH terminology to better reflect the hospital-level care provided at home, align with global standards and reduce confusion amongst patients [14]. Although much of the recent literature refers to VWs, this paper follows BGS’s terminology recommendation for clarity and refers to all models as Hospital at Home (HaH).

The main HaH models include ‘Step-up’ hospital admission avoidance and ‘Step-down’ early hospital discharge, with subsequent home treatment and monitoring [15–17]. Studies show that these models offer safety and clinical effectiveness comparable to hospitalisation, alleviating staff and healthcare system pressures [15–18]. However, existing reviews focus on quantitative outcomes like mortality and readmissions, often neglecting components that optimise outcomes from the perspectives of patients, family carers (hereafter carers) and healthcare professionals [15, 17, 18]. A research agenda established after the First World HaH Congress in 2019 identified HaH experiences as a key priority for understanding barriers and facilitators to home care [19].

The aim of this review was to systematically examine the literature of HaH care for older people in the UK, focussing on older people, carers and healthcare professionals’ experiences and perceptions of what optimises outcomes.

Objectives include

1. Summarise HaH experiences and perspectives of older people, carers and healthcare professionals.
2. Understand service and patient-level barriers and facilitators for optimising care outcomes.
3. Compare different HaH models and care delivery to support older people at home.
4. Devise recommendations for clinical practice and policy to optimise HaH care for older people.

## Methods

This systematic review was conducted in accordance with the Preferred Reporting Items for Systematic reviews and Meta-Analyses (PRISMA) 2020 statement [20] (see Appendix 1). This was also used to shape the search strategy and eligibility criteria using the Population, Intervention, Comparator, Study Type approach [20]. Searches were conducted up to January 2024 (PROSPERO protocol ID: CRD42024535878).

### Eligibility criteria

This review focused on the UK due to its unique publicly funded healthcare and social care system, which integrates HaHs into this framework. This approach allows for a targeted understanding of factors influencing care that are directly relevant to national policy and practice, enabling incorporation of UK-specific grey literature.

### Search strategy

This was an iterative process with search terms devised in collaboration with an information specialist and Patient Public Involvement (PPI) members to ensure an extensive search (see Table 1). An initial scoping search of literature was conducted on MEDLINE using the concepts of ‘virtual ward’ OR ‘hospital at home’ AND ‘older adult’ to develop keywords and Medical Subject Headings (MeSH) terms. A comprehensive search on MEDLINE, CINAHL and EMBASE was undertaken in January 2024 using the terms identified from the scoping search (search strategy results seen in Appendix 2). Grey literature was searched in March 2024 from Overton (NHS and government documents), customised Google search engines (first 10 pages) and think tanks (The Kings Fund and Nuffield Trust).

**Table 1:** Inclusion and exclusion criteria.

	Inclusion	Exclusion
**Population**	▪ Older adults, >65 years of age ▪ Studies with age ranges only considered if >50% of participants were >65 or if separate analysis of this age group was clearly reported ▪ Staff and carers aiding older adults on HaH	▪ (Majority) <65 years, with no separate analyses of >65
**Intervention**	▪ Virtual Ward or Hospital at Home care as the intervention for older adults ▪ Within the UK	▪ Psychiatric, palliative, paediatric and nursing home HaH models ▪ Transitional care models (moving patients from hospital to home) as focus is on physical relocation from hospital to home rather than ongoing management ▪ Specific treatments as primary interventions ▪ Primary focus or intervention was not HaH such as occupational therapy programmes or integrated community services ▪ Not in the UK
**Comparator**	N/A	N/A
**Outcome**	▪ Description of care models ▪ Perspectives/experiences of family carers, older adults or staff ▪ Barriers and facilitators to delivery of virtual wards	
**Study type**	▪ All types of qualitative and quantitative primary study designs ▪ Published in English	▪ Literature reviews ▪ Case studies ▪ Letters ▪ Commentaries

N/A, not available.

### Screening and selection process

Titles and abstracts were screened against the inclusion and exclusion criteria using the Rayyan Systematic Review Software by one reviewer (A.W.). A random 10% of these were double screened by a second reviewer (C.S.A.). Any disputes were resolved via discussion and consensus or, if necessary, involvement of a third reviewer (N.D.). This process was repeated in the full-text screening. Subsequent forward and backward citation tracking of included full texts were conducted.

### Data extraction

A data extraction tool was developed in Microsoft Excel, informed by the Cochrane data extraction template—seen in Appendix 3 [21]. This was completed by one reviewer (A.W.) and a random 20% checked by a second reviewer (N.D.).

**Table 2:** Study characteristics and key findings.

Author, year	Study type	Methods	Population characteristics: (total)	Population breakdown	Mean patient age (years)	Key findings
Chen, 2024 [22]	Literature review + qualitative	Small group/individual interview	Staff: 16	5 nurses, 8 doctors. 1 PA, 1 PT, 1 OT	Services were mostly older people >65	Holistic healing effects of home environment, better therapeutic staff–patient–carer relationships, more continuity of care
Dismore, 2018 [23]	Qualitative embedded in RCT	Semi-structured interview and Carer Burden Scale	Patient: 31 Carer: 15 Staff: 30	Patient: (15 HaH, 16 hospital), 13 decliners Carer: (10 HaH, 5 hospital) Staff: 11 specialist nurses, 15 doctors, 4 managers	*68* Decliners: *73*	HaH preferred—more independence, maintenance of daily routine, better sleep. Safety concerns of patient being alone at night
Dowell, 2018 [24]	Qualitative	Telephone questionnaire	Patient, carer: 105 Staff: GPs	N/A	*84% 65 to 85+*	High satisfaction. HaH allows for holistic care
Gunnell, 2000 [25]	RCT	Postal Questionnaire—carer strain index, patient satisfaction	Carer: 133	93 HaH, 40 hospital	65	HaH had no significant impact on carer burden, HaH carers had significantly higher carer satisfaction compared to hospital carers
Health Innovation Network, 2021 [26]	Grey literature—mixed methods	Current Health Patient Experience Survey and Interview	Patient Questionnaire: 37 Patient Interview: 3	N/A	60% of patients >60, 25% of patients >80	Therapeutic care relationship enabled shared decision-making, increased patient/carer confidence. Staff recognise digital exclusion
Jester, 2002 [27]	Mixed methods—longitudinal follow-up study	Modified hospital patient satisfaction index survey and carer semi-structured interviews	Patient: 109 Carer: 21	Patient: 64 HaH, 45 hospital	74	Patient satisfaction significantly higher in HaH vs hospital. All but one carer would choose HaH care again
Karacaoglu, 2021 [28]	Qualitative	Satisfaction questionnaire and semi-structured interview	Staff: 13	3 Advanced practitioners, 5 healthcare support workers, 2 pharmacists, 3 management	86.2	Upskilling care practitioners seen as facilitator. Positive feedback from patients regarding increased confidence, and value of home support
Kirkcaldy, 2017 [29]	Qualitative	Focus group interview	Staff: 14	5 pharmacists, 9 from wider MDT (PT, OT, district nurses)	N/A due to staff perspectives	Increased patient confidence at home, value of HaH holistic care. Challenge accessing patient GP record by pharmacist
Knowelden, 1991 [30]	Qualitative	Questionnaire—degree of satisfaction and interview of carers	Patient questionnaire: 105 Patient interview: 66	Questionnaire: 50 HaH, 55 hospital Interview: 33 HaH, 6 with carer*,* 33 hospital	HaH: 67 Hospital: 64	Patients content with HaH care. Carers found some degree of burden with HaH but was similar with burden from usual hospital care
Kotb, 2023 [31]	Qualitative—proof-of-concept study	Questionnaire—NHS Friends and Family test and narrative feedback	Patient: 45	N/A	66	Feelings of empowerment, active care participation, hospital stay avoidance and ease of access to healthcare staff. Difficulty with technology, lack of communication and lack of ‘visible’ healthcare staff
Makela, 2020 [32]	Qualitative—within RCT	Semi-structured interview	Patient: 34 Carer: 29	Patient: 15 HaH*,* 19 hospital Carer: 12 HaH, 17 hospital	HaH: 83 Hospital: 84	Carers facilitate HaH continuity of care. Upskilling healthcare professionals facilitate care. Barriers: lack of shared decision-making, and HaH safety concerns
Ojoo, 2002 [33]	RCT	Structured interview of satisfaction questionnaire	Patient: 54 Carer: 34	Patient: 27 HaH, 27 hospital	HaH: 69.7 Hospital: 70.1	Patients and carers prefer HaH
Saleh, 2024 [34]	Qualitative—service evaluation	Questionnaire—NHS Friends and Family Test	Patient: 43	N/A	65	Remote monitoring equipment increased patient perceptions of safety. Increased empowerment via digital education. Barriers: technical difficulty, digital exclusion
Schiff, 2022 [35]	Qualitative	Retrospective telephone questionnaire	Patient: 3 Carer: 13	N/A	85	Increase NHS capacity for beds, value of staying at home with family support
Schofield, 2005 [36]	Mixed methods	Postal survey + interview	Patient, carer: 104	All used HaH service Total interviews: 30 (18 patient, 10 patient and carer, 2 carer)	68	Wholly positive experiences with HaH compared to negative hospital experiences
Shepperd, 1998 [37]	RCT	Satisfaction questionnaire Carer strain index to measure carer burden	Patient: 347 Carer: 155	Patient: 149 HaH, 198 hospital Carer: 80 HaH, 75 hospital	Mean age ranged depending on pathway HaH: *68–77* Hospital: 70–76	All patients except those with COPD preferred HaH care. No significant differences in carer burden between HaH and hospital
Shepperd, 2021 [38]	Multi-site RCT	Picker Institute patient-reported experience questionnaire	Patient: 1032	687 HaH, 345 hospital	83.3	Responses regarding wait time, how to contact staff and decision-making involvement favoured HaH
Thornton, 2023 [39]	Grey literature	Survey from census	Public: 7100 Staff: 1251	N/A	>16 years, included 65+ group	Public and NHS staff support HaHs, further need to clarify HaH terminology
Vindrola-Padros, 2021 [40]	Qualitative	Semi-structured interview	Staff: 22	8 pilot site leads, 7 monitoring leads, 7 staff with knowledge of data collection	N/A due to staff perspectives	Personalised care. Barriers: digital exclusion, lack of culturally appropriate resources, poor integration of service data with existing administration systems
Wilson, 2002 [41]	RCT	Questionnaire + semi-structured interview	Patient questionnaire: 83 Patient interview: 42 Carer: 25	Questionnaire: 48 HaH, 35 hospital Patient interview: 24 HaH, 18 hospital Carer interview: 18 HaH, 7 hospital	Paper reported median age HaH: 82 Hospital: 81	HaH more personalised, better communication vs hospital. Value of home. Carers had safety concerns. No carer burden increase

N/A, not available; OT, occupational therapist; PA, physician’s associate; PT, physiotherapist; RCT, randomised controlled trial.

### Quality appraisal

The Hawker critical appraisal tool was used to appraise each paper by one reviewer (A.W.) and a random 20% checked by a second reviewer (N.D.) [42]. They were scored from 1 to 4 on nine items to give a total score. These scores were categorised as low (total score 9–24), medium (total score 24–29) or high quality (total score 30–36) indicated by the colours red, orange and green in Appendix 4. The AACODS checklist (Authority, Accuracy, Coverage, Objectivity, Date and Significance) was used for grey literature appraisal [43].

### Data synthesis

A stepwise narrative synthesis model was utilised, guided by Popay *et al*. [44, 45]. This included tabulation of studies in thematic analysis form and concept mapping. Each theme was plotted and lines drawn to represent relationships between them (positive and/or negative experiences identified by study participants). Barriers and facilitators to specific themes were included where relevant.

### Patient and public involvement and stakeholder involvement

Two former family carers of older people guided the methods of this review. We hosted a meeting to gather feedback on the themes and potential key messages for recommendation.

## Results

### Study selection

**Figure 1:**
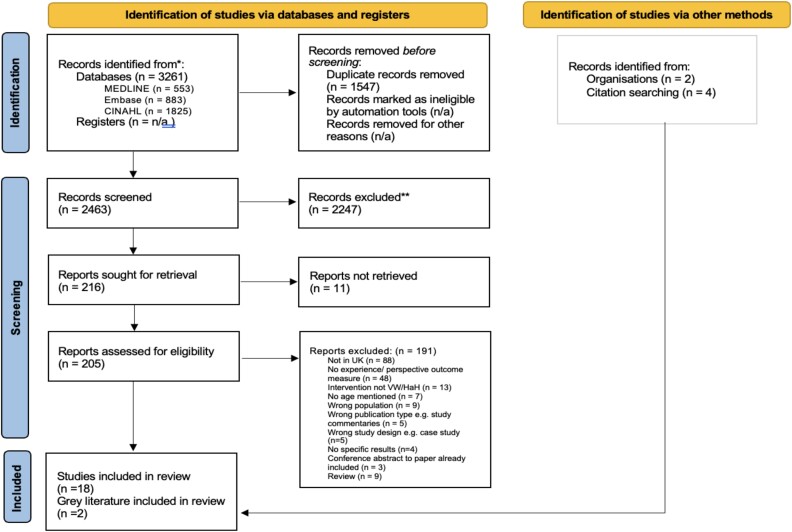
PRISMA flow of study selection [20].

After removing duplicates, 2463 records were identified and assessed for relevance at title and abstract level. Also, 205 were assessed at full text, leading to the inclusion of 18 studies [22–25, 27–38, 40, 41] (see Table 2 for details). Grey literature database searches yielded 674 records with two included in this review [26, 39]. Figure 1 includes reasons for exclusion at full-text eligibility stage [20].

### Study characteristics

Of the included papers, 11 were qualitative [23, 24, 28–32, 34, 35, 39, 40], 5 were randomised controlled trials [25, 33, 37, 38, 41] and 4 were mixed methods [22, 26, 27, 36]. The most common perspectives within included studies were those of patients and family carers (*n* = 1349 total participants, with *n* = 256 carers within this) [27, 30, 32, 33, 35–37, 41]. A further four studies included patient, carer and healthcare professional perspectives [23, 24, 26, 39].

### Quality appraisal

The included studies were of moderate-good quality, with only two studies rated as poor. All included studies had a clear description of methods, population characteristics, data collection and justification of sample sizes, with findings in line with stated aims. Studies were rated poorly due to brief acknowledgement, if at all, of authors’ bias and limited generalisability to the wider population—seen in Appendix 4.

### Care models

Studies report ‘step up’ admission avoidance [24, 26, 29, 32, 38, 41], ‘step down’ early discharge [23, 25, 27, 28, 30, 33, 36, 37] or both models [22, 31, 34, 35, 39, 40]. Most HaH services operated 7 days a week—four studies had 24-h service models [23, 24, 37, 41]. All had signposting to HaH staff contact numbers or out-of-hours services [23, 24, 26, 29, 31, 34, 38, 40]. Levels of digital technology implementation varied, but all included remote care via telephone calls and face-to-face home visits by healthcare professionals. Remote monitoring applications had safety mechanisms notifying patients and healthcare teams when recorded data indicated potential deterioration, signposting contact information for assistance [26, 34, 40].

Included studies evaluated condition specific HaHs (most commonly COPD, atrial fibrillation and COVID-19) [23, 31, 33–35, 40, 46, 47]. Studies also looked at general HaHs led by consultant geriatricians or other healthcare professionals such as specialist nurses or advanced nurse practitioners [22, 24, 25, 27–30, 32, 37, 39, 48]. Conditions treated include frailty, dementia, delirium, stroke, post-surgery recovery from hip or knee replacements, urinary tract infections and falls (Table 3).

**Table 3:** HaH service models.

Author, year	Clinical responsibility	Healthcare professionals’ team	Conditions treated	HaH model	Technology enabled?^a^
Chen, 2024 [22]	Consultant geriatrician/community specialist nurse	Nurse, ANP, SN, ACP, consultant geriatrician, GP, OT, PT, speech therapist, pharmacist, administration staff, PA	Unspecified—‘older patients with a range of conditions’	Admission avoidance and early discharge Home visits and telephone calls by staff	No
Dismore, 2018 [23]	Respiratory consultant	Respiratory specialist nurse, respiratory consultant, GP	COPD	Early discharge, nurse led Home visits 1–2× daily by nurses	No
Dowell, 2018 [24]	Paramedics, nurses and physiotherapists	Nurse, GP, PT, specialist paramedic	Unwell adult pathway, COPD, UTI, palliative, IV, cellulitis, falls	Admission avoidance, 24 h Referral triage system	No
Gunnell, 2000 [25]	Not stated	Not stated	Fracture, elective orthopaedic surgery, stroke, dementia	Early discharge	No
Health Innovation Network, 2021 [26]	2 consultant geriatricians, 1 respiratory consultant, rapid response GP	Nurse, consultant geriatrician, respiratory consultant, GP, project lead, rapid response matron	COVID-19	Admission avoidance, 8 a.m.–8 p.m., 7 days a week Staff contacted patient via phone/video call, home visits. Out-of-hours service (111/999) Wearable integrated with tablet. Home hub connects wearable to cloud. Vital signs continuously collected and displayed on web dashboard for remote monitoring team	Yes
Jester, 2002 [27]	Orthopaedic surgeon	Nurse, orthopaedic consultant, PT	Hip/knee replacement surgery recovery	Early discharge, home visits from nurse/PT at least 1× a day. 7 days a week, 8 a.m.–8 p.m. Out of hours: senior on-call nurse	No
Karacaoglu, 2021 [28]	Clinical guidance from consultant geriatrician	ANP, consultant geriatrician, OT, PT, pharmacist, healthcare support worker	Geriatric syndrome	Early discharge providing rehabilitation support and home visits from nursing, PT, OT	No
Kirkcaldy, 2017 [29]	Not stated	Nurse, OT, PT, pharmacist, social care, community matron	Unspecified ‘older patients with a range of conditions’	Admission avoidance—has medicines management team that delivers support to increase adherence	No
Knowelden, 1991 [30]	GP	Nurse, GP	Metastatic neoplasm, early discharge post-surgery, stroke	Early discharge, mainly district nurse led with home visits	No
Kotb, 2023 [31]	Consultant cardiologist	Nurse, ACP, cardiology consultant, registrar, pharmacist	Atrial fibrillation, atrial flutter and fast ventricular response	Admission avoidance, early discharge. 7-days-per-week service, 9 a.m.–5 p.m. Out of hours: on-call cardiology registrar/emergency services Provided with ECG devices, Bluetooth blood pressure monitor, pulse oximeter, smartphone app to record readings/symptom severity. If readings exceed threshold, patients and healthcare team notified. In-app messaging, telephone, video calls	Yes
Makela, 2020 [32]	Geriatrician	ANP, consultant geriatrician, GP, speciality training doctor, OT, PT, speech therapist, pharmacist, social care	Falls, delirium, COPD, back pain, cellulitis, chest infection, other	Admission avoidance—7 days a week, 9 a.m.—early evening. Emergency medical cover available 24 h a day. Primary care support	No
Ojoo, 2002 [33]	Respiratory outreach nurse	Respiratory specialist nurse	COPD	Early discharge—9 a.m.–5 p.m., monitor patients daily. Out-of-hours services—Medical Chest Unit direct line	No
Saleh, 2024 [34]	Cardiology consultant	Nurse, digital technology specialist nurse, ANP, cardiology consultant, registrar, allied healthcare professionals	Atrial fibrillation	Admission avoidance, early discharge. 8 a.m.–8 p.m. Out-of-hours signposting to 111/999 Weekly MDT meetings to discuss HaH patients FIBRICHECK remote monitoring app. Measurements and symptom severity recorded into app twice daily. Monitored by hub, where a dashboard of patients’ clinical data could be reviewed by clinicians twice daily	Yes
Schiff, 2022 [35]	Not stated	Not stated	COVID-19	Admission avoidance, early discharge	No
Schofield, 2005 [36]	Respiratory consultant	Respiratory specialist nurse, respiratory consultant	COPD	Early discharge, 9 a.m.–5 p.m. Respiratory nurses do home visits and outreach treatment	No
Shepperd, 1998 [37]	GP	ANP, doctors, allied health professionals	Hip/knee replacement recovery, older medical patients, COPD	Admission avoidance, early discharge. Home visits, observations, administration of drugs such as IV, rehabilitation Patients provided mobile phones if needed	No
Shepperd, 2021 [38]	Geriatrician	Nurse, consultant geriatrician, speciality training doctor, OT, PT, speech therapist, pharmacist, social care	Dementia, falls, respiratory. gastrointestinal, musculoskeletal disorders, cardiovascular conditions, UTI	Admission avoidance—7 days a week, some sites offered 24-h care, but most were 9 a.m. to early evening. Emergency medical cover available 24 h a day. Primary care support	No
Thornton, 2023 [39]	Not stated	Nurse, doctors	Unspecified	Admission avoidance, early discharge Technology for monitoring considered in definition of HaH	Yes
Vindrola-Padros, 2021 [40]	GP/consultant depending on site	Nurse, ANP, respiratory specialist nurse, consultant, GP, registrar, senior and junior clinicians, PT, pilot site lead, OPAT nurse, practice manager, PA	COVID-19	Admission avoidance, early discharge. Regular monitoring calls from primary or secondary care staff. Provided with pulse oximeter, digital app or paper diary to record observations. If patient observation exceeded ‘safe threshold’, patients and clinical team notified	Yes
Wilson, 2002 [41]	GP	Nurse, GP, OT, PT, generic health worker	Cardiovascular and respiratory conditions	Admission avoidance, 4–24-h care. Home visits by nurses	No

*Note*: Gunnel *et al*. did not specify specific staffing in HaH model. ANP, advanced nurse practitioner; ACP, advanced clinical practitioner; OPAT, outpatient parenteral antimicrobial therapy; OT, occupational therapist; PA, physician’s associate; PT, physiotherapist; SN, nurse specialist; UTI, urinary tract infection.

^a^NHS definition of technology-enabled HaH consists of the following criteria [49]:

1. Patients measuring and inputting their health data into an application/done automatically via wearable or Bluetooth.

2. Data feeds into digital platform where the clinical team can review.

3. Clinical teams are alerted when patient moves outside of safe parameters, and can act accordingly.

### Perceptions of virtual wards

**Figure 2:**
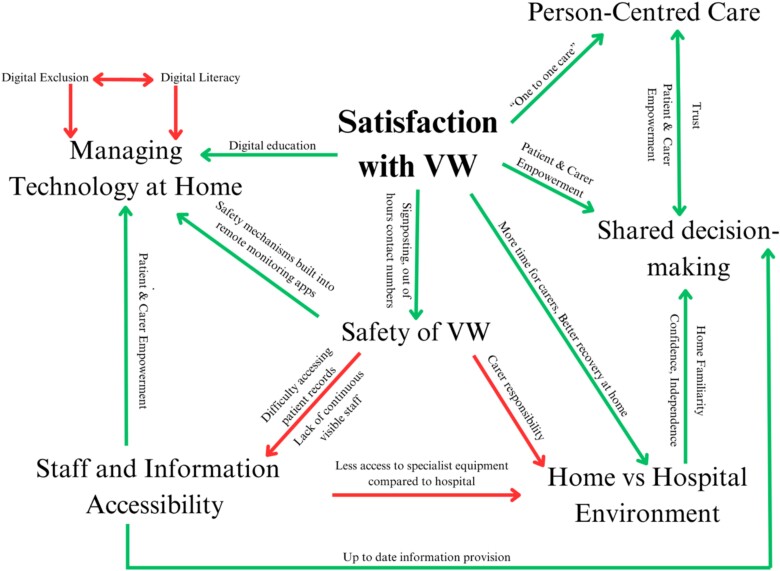
Concept map of key themes, barriers and facilitators.

Six themes were generated from included studies and are represented in a concept map detailed in Figure 2.^1^

#### Familiarity of home versus a hospital environment

Studies highlighted home being the most comfortable environment for recovery due to better social support, sleep, nourishment and stress levels [22, 23, 27, 32, 35, 36, 41]: ‘You’re in your own surroundings which helps you get better quicker’ (HaH patient) [41]. Home familiarity in HaHs was found to positively influence patient confidence and independence, with HaH staff reporting their role in building this confidence during home assessment [22, 23, 25, 26, 32, 35, 37, 41]. It also helped mitigate distress for patients experiencing acute confusion and delirium due to unfamiliar hospital surroundings [22, 32]. Patients and carers across studies appreciated not being separated from their family [23, 24, 27, 35, 41]: ‘Having his family around him was the most important thing for him, and Hospital at Home allowed that to happen’ (Relative to COVID-19 HaH patient) [35].

Common negative hospital experiences described were in regard to poor cleanliness, nutrition and sleep disruption due to noise, nightly observations and lack of privacy [23, 33, 36, 41]. Carers faced challenges related to travel, restrictive visiting hours, absence of familial support and parking costs, making hospital stays less favourable [23, 27, 41]. However, it was not all negative for hospitals, as there was a balance to be struck between the value of the home environment versus the timely access to specialist care and equipment offered in hospital [35, 41].

#### Person-centred care

Person-centred care delivered by the HaH team was consistently recognised as a strength of care delivery by patients, carers and staff. HaH nurses were frequently commended for their clear explanations of treatment plans, active listening and rapport-building—often described to be ‘friendly’, ‘respectful’, ‘approachable’, ‘excellent’ and ‘caring’ [23–27, 30, 33, 34, 36, 38, 41]. Patients and carers especially appreciated the ‘one-to-one’ care relationship with nurses [23, 26, 41]: ‘I think you got more attention… it seems as if you are the only one…’ (HaH patient) [41]. Studies described similar or reduced carer burden, and higher carer satisfaction scores with HaHs compared to hospital [22, 25–27, 35, 37, 41]. Several frail family carers reported appreciation for the attention they received from HaHs: ‘…the home care nurses, they couldn’t have done more for him… and they looked after me.’ (HaH family carer) [41].

#### Enabling shared decision-making

Staff were identified to provide well-informed and up-to-date information on patient progress, particularly on discharge plans, additional equipment needs, home adaptations and service referrals [24, 25, 30, 31, 33, 36, 38, 39]. They enabled shared decision-making by eliciting patient and carer ideas and concerns regarding HaH care delivery [24, 25, 30, 31, 33, 36, 38, 39]: ‘It gives us a chance to be more involved in treatment and understanding the problem…’ (Patient onboard an atrial fibrillation HaH) [31]. Within papers that scored participants’ experiences, HaH patients and carers reported higher levels of satisfaction with discussions with healthcare staff and involvement in care decisions compared to usual hospital care groups [25, 38]. This was often contrasted with previous patient and carers’ lack of involvement in decision-making in hospital settings, where perceptions of NHS staff ‘rushing to empty beds’ meant care was transactional and less personalised compared to HaHs [32].

#### Challenges with staff and information accessibility

Family carers reported instances where HaH staff should have visited more frequently, with a lack of continuous ‘visible’ staff impacting their perceptions of HaH effectiveness [27, 31]. Makela *et al*. identified the role of carers acting as a ‘bridge to continuity of healthcare’ and facilitating HaH care [32]. Dissatisfaction with HaH care delivery arose when patients and carers reported difficulty contacting staff and obtaining patient records and goals set by the HaH team [27, 31–33]. The absence of written communication summaries reduced clarity for patients and carers post-discharge from HaHs, describing these as a form of communication between professionals rather than for patients [31, 32, 40].

Karacaoglu *et al*. and Vindrola-Padros *et al*. raised the challenges in delivering a 7-day service due to clinical lead unavailability, challenges with recruiting senior clinicians or having sufficient cover for HaHs to operate [28, 40]. Allied healthcare professionals and pharmacists cited difficulty in accessing GP patient health records [29]. Integrating HaH patient data with primary care record systems like EMIS and expansion of referral pathways to more acute departments were recognised facilitators for continuity of care in future practice [26, 28, 32].

#### Safety of HaH

Patient and family carers recognised safety as a priority in hospitals and raised concerns that HaHs required increased carer involvement and responsibility in monitoring patients, highlighting patient vulnerability at night when HaH staff were not present [22, 23, 27, 32, 41]: ‘it’s like sleeping with one eye open…’ (Family carer) [32].

However, some patients, especially those living alone, valued the provision of accessible telephone numbers, nurses’ encouragement to call with concerns and scheduled evening phone calls from healthcare professionals [22, 23, 41]:‘They all seemed to me to be very well trained and put me at ease’ (Patient in COPD HaH) [23].

#### Managing technology at home

Older people and their carers faced difficulty with HaH technology, including concerns about personal data privacy and patient anxiety or aversion to technology [26, 31, 34, 39, 40]. Staff concerns focused on digital exclusion as a barrier to HaH implementation, highlighting the need for a minimum level of patient digital literacy and access to devices for monitoring applications [26, 39, 40]. Saleh *et al*. and Vindrola-Padros *et al*. noted a lack of culturally appropriate resources in different languages, contributing to digital exclusion for non-English speakers [34, 40].

Despite this, many older people and their carers responded positively to digital technology [26, 31]*.* A key facilitator for HaH care delivery was the promotion of digital health education and clear explanations from healthcare professionals to build patient confidence in using technology [26, 31, 34, 39, 40]. Flexibility in equipment to enhance digital inclusion was demonstrated by using physical pulse oximeters for patients without compatible smartphones [34].

## Discussion

Our findings indicate that person-centred care, rapport-building, home familiarity and shared decision-making are key components of HaHs from the perspectives of patients and family carers. Concerns included staff availability, digital literacy and carers’ anxiety about managing care, especially at night, without the reassurance of in-person HaH staff support. A summary of positive and negative themes with recommendations are provided in box 1.

Box 1. Summary of positive and negative themes with recommendations.

**Positive themes** 

- Improved wellbeing and independence at home

- Person-centred, ‘one-to-one’ care

- Strong patient–staff relationship

- HaH nurses identifying frail family carers

- Staff keeping patients and carers well informed on progress, treatment and discharge plans, enabling shared decision-making

- Clear explanations from staff to empower patients with technology use

**Negative themes with recommendations** 

- Lack of staff during the night

- Recommendation: Scheduled evening phone calls with HaH staff, centralised overnight support service

- HaHs required increased carer responsibility in monitoring patients

- Recommendation: Use wearable devices that automatically send patient data to healthcare teams, provide comprehensive training programmes for carers on how to recognise warning signs, establish carer support services

- Carers found difficulty contacting staff and obtaining patient records

- Recommendation: Clear signposting of staff contact numbers, implementing cloud-based systems for real-time updates and syncing of patient records across teams

- Difficulty accessing GP patient health records between teams

- Recommendation: Integrate HaH patient data with other health record systems, expansion of referral pathways to more acute departments

- Digital exclusion of older adult patients

- Recommendation: Digital health education with patients and carers to build confidence, providing alternative equipment for those without compatible smartphones

### Care environments

The therapeutic value of receiving care in a familiar home environment was a key driver for patient confidence, independence and recovery, especially for older people with dementia [22, 23, 27, 32, 35, 36, 41]. Despite views that hospital provided more specialised care and equipment compared to HaHs, there was a strong feeling from patients and carers regarding ‘disruptive hospital’ environments, reinforcing overarching preferences and satisfaction with home-based HaH care [23, 35, 36, 41].

Home supports the preservation of self-identity in older people, rather than reducing them to the role of a patient [50, 51]. Recognition of the benefits of a ‘small homelike environment’ for older people with dementia aligns with patient and carer preferences cited in this review [52–55]. Harreman *et al*. conceptualises ‘ageing in place’ as the process of a house becoming more than a physical space, deeply connected to personal identity [56]. This supports HaH care models, as it allows older people to preserve their autonomy, whilst maintaining the relationships that are central to their sense of self [50, 51, 57, 58].

### Availability of family carer support

This review reveals a significant lack of carer representation in rigorous HaH studies, highlighting a research gap in the support and involvement of family carers. Although carer views were included and highly valuable for analysis in this review, the total number of carers participating was significantly lower than that of patients. This is an important omission, as the experiences of carers may be very different to care recipients. This difference in research participation may be due to carer-specific barriers such as a lack of time, managing their own health problems and the challenges of balancing active research involvement with their caring responsibilities [59].

### Use of technology with older people

The UK Office of National Statistics consistently show older people as the largest proportion of adult internet non-users [60]. This age-related digital divide contributes to older adult hesitancy of HaH implementation and is further exacerbated by age-related barriers such as cognitive decline and physical impairments making technology use difficult [61–64]. Although the atrial fibrillation and COVID-19 HaHs in this review tailored care strategies to promote digital inclusion, there was no discussion of how to accommodate individuals with cognitive impairment using these technologies [26, 31, 34, 40].

Public perceptions of equating HaH care delivery with solely ‘virtual consultations’ may discourage older people who typically prefer face-to-face care [39]. However, since COVID-19, the increase in teleconsultation adoption amongst older people highlights a significant shift in digital engagement and potential for sustained digital integration within this demographic [65].

### Implications and recommendations to practice, policy and research

Our findings highlight the importance of patient, carers and healthcare professional’s perceptions in optimising HaH care delivery. Future research should include more diverse perspectives from these stakeholder groups to align HaH models with user and provider needs.

Clear summaries of HaH care pathways can aid with clarifying HaH terminology for older people to enhance acceptance and understanding. Meanwhile, standardising communication protocols amongst healthcare providers can enable better information sharing. Policy initiatives should prioritise culturally appropriate digital care to increase digital education and equitable access to HaHs, such as simplified interfaces or supportive web platforms [66, 67].

Public Health England’s rapid review identified unpaid caring as a social determinant of health and a need for higher quality estimates of carer burden [68]. This mirrors gaps in HaH carer support services, indicating the need for greater carer inclusion in research to develop evidence-based systems that ease carer burden [69, 70]. There may be value in implementing systems to provide and signpost family carers to services that address their physical and emotional needs during their time caring for individuals onboard HaHs. Whilst not within the scope of this review, UK HaH systems can draw useful insights and apply study findings from global models in the Netherlands, USA, Singapore and Australia that focus on family carer strain experiences, thus enriching UK research on these outcomes [71–76].

### Strengths and limitations

This review uses a broad systematic search strategy. A key strength is the inclusion of carer representation via PPI, amplifying often underrepresented carer voices and enhancing the relevance of findings and recommendations [77]. Only studies published in the UK and in English were included, so findings may not be applicable to other settings; however, the key messages may be considered in similar systems and settings to those in the UK. Quality appraisal revealed limited ethnic diversity in participant demographics, limiting representation of their experiences. Inconsistencies of how HaHs are defined and labelled across studies may have overlooked relevant results that do not include the terms ‘Virtual Ward’ or ‘Hospital at Home’, but the iterative development and broad variety of terms in the search potentially mitigates this risk. As this paper focusses on different conditions across the HaH models, the experiences on each might not be universally applicable. However, the core themes presented such as familiarity in the home environment, carer burden and provision of person-centred care should overlap between care models.

## Conclusion

From patient, carer and healthcare professional perspectives, the value of care provision at home for older people embodies autonomy, dignity and faster recovery. However, empowerment is key to facilitate older people’s digital engagement. HaHs allow for a holistic approach in addressing older people’s care needs, with potential support for carers too. Carers play a significant role in providing a link between HaH care and older people. Yet, it is unknown how to best implement carer support; therefore, future studies should focus on devising sustainable carer support systems within HaHs and gather their perspectives to further optimise patient, carer wellbeing and satisfaction with HaH services.

## Supplementary Material

aa-24-2318-File002_afaf033

## Footnotes

1 All themes stem from general satisfaction with HaHs. The green arrows represent positive perspectives and experiences, highlighting facilitators and the link between themes. For example: the familiarity of home environment facilitates shared decision-making as older people and family carers are more confident and comfortable at home. The red arrows highlight concerns and barriers in relation to and between themes. For example: experiences of increased carer responsibility led to their anxiety at night when the HaH staff were not present are barriers to HaH safety. The double-headed arrow represents the mutual influence between person-centred care and shared decision-making within HaHs.
