# Supplementary material for: Supporting older people through Hospital at Home care: a systematic review of patient, carer and healthcare professionals’ perspectives
Source: Age Ageing. 2025 Feb 23;54(2):afaf033. doi: 10.1093/ageing/afaf033 (PMC11847509; doi:10.1093/ageing/afaf033)
Supplement: aa-24-2318-File002_afaf033 [file aa-24-2318-file002_afaf033.docx]

# **Supporting older people through Hospital at Home care: A systematic review of patient, carer, and healthcare professionals’ perspectives**

**Supplementary Materials:**

Appendix 1: PRISMA Checklist

Appendix 2: Search Strategy

Appendix 3: Data Extraction Topics

Appendix 4: Quality Appraisal of literature

Appendix 5: Summary table of quotes for each theme

Appendix 1: PRISMA Checklist

| **Section and Topic** | **Item #** | **Checklist item** | **Location where item is reported** |
| --- | --- | --- | --- |
| **TITLE** | | |  |
| Title | 1 | Identify the report as a systematic review. | p1 |
| **ABSTRACT** | | |  |
| Abstract | 2 | See the PRISMA 2020 for Abstracts checklist. | p2 |
| **INTRODUCTION** | | |  |
| Rationale | 3 | Describe the rationale for the review in the context of existing knowledge. | p2,3 |
| Objectives | 4 | Provide an explicit statement of the objective(s) or question(s) the review addresses. | p3 |
| **METHODS** | | |  |
| Eligibility criteria | 5 | Specify the inclusion and exclusion criteria for the review and how studies were grouped for the syntheses. | p4 Table 1 |
| Information sources | 6 | Specify all databases, registers, websites, organisations, reference lists and other sources searched or consulted to identify studies. Specify the date when each source was last searched or consulted. | P4, 5 |
| Search strategy | 7 | Present the full search strategies for all databases, registers and websites, including any filters and limits used. | p5 |
| Selection process | 8 | Specify the methods used to decide whether a study met the inclusion criteria of the review, including how many reviewers screened each record and each report retrieved, whether they worked independently, and if applicable, details of automation tools used in the process. | p5 |
| Data collection process | 9 | Specify the methods used to collect data from reports, including how many reviewers collected data from each report, whether they worked independently, any processes for obtaining or confirming data from study investigators, and if applicable, details of automation tools used in the process. | p5,6 |
| Data items | 10a | List and define all outcomes for which data were sought. Specify whether all results that were compatible with each outcome domain in each study were sought (e.g. for all measures, time points, analyses), and if not, the methods used to decide which results to collect. | p5 |
|  | 10b | List and define all other variables for which data were sought (e.g. participant and intervention characteristics, funding sources). Describe any assumptions made about any missing or unclear information. | p5 |
| Study risk of bias assessment | 11 | Specify the methods used to assess risk of bias in the included studies, including details of the tool(s) used, how many reviewers assessed each study and whether they worked independently, and if applicable, details of automation tools used in the process. | p5 |
| Effect measures | 12 | Specify for each outcome the effect measure(s) (e.g. risk ratio, mean difference) used in the synthesis or presentation of results. | n/a |
| Synthesis methods | 13a | Describe the processes used to decide which studies were eligible for each synthesis (e.g. tabulating the study intervention characteristics and comparing against the planned groups for each synthesis (item #5)). | p5, 8-10 Table 2 |
|  | 13b | Describe any methods required to prepare the data for presentation or synthesis, such as handling of missing summary statistics, or data conversions. | n/a |
|  | 13c | Describe any methods used to tabulate or visually display results of individual studies and syntheses. | p5 |
|  | 13d | Describe any methods used to synthesize results and provide a rationale for the choice(s). If meta-analysis was performed, describe the model(s), method(s) to identify the presence and extent of statistical heterogeneity, and software package(s) used. | p5,6 |
|  | 13e | Describe any methods used to explore possible causes of heterogeneity among study results (e.g. subgroup analysis, meta-regression). | n/a |
|  | 13f | Describe any sensitivity analyses conducted to assess robustness of the synthesized results. | n/a |
| Reporting bias assessment | 14 | Describe any methods used to assess risk of bias due to missing results in a synthesis (arising from reporting biases). | n/a |
| Certainty assessment | 15 | Describe any methods used to assess certainty (or confidence) in the body of evidence for an outcome. | p5 |
| **RESULTS** | | |  |
| Study selection | 16a | Describe the results of the search and selection process, from the number of records identified in the search to the number of studies included in the review, ideally using a flow diagram. | p5-7 |
|  | 16b | Cite studies that might appear to meet the inclusion criteria, but which were excluded, and explain why they were excluded. | n/a |
| Study characteristics | 17 | Cite each included study and present its characteristics. | p8-10 Table 2 |
| Risk of bias in studies | 18 | Present assessments of risk of bias for each included study. | Appendix 4 |
| Results of individual studies | 19 | For all outcomes, present, for each study: (a) summary statistics for each group (where appropriate) and (b) an effect estimate and its precision (e.g. confidence/credible interval), ideally using structured tables or plots. | p8-10 Table 2, p12-14 Table 3, |
| Results of syntheses | 20a | For each synthesis, briefly summarise the characteristics and risk of bias among contributing studies. | p8-10 |
|  | 20b | Present results of all statistical syntheses conducted. If meta-analysis was done, present for each the summary estimate and its precision (e.g. confidence/credible interval) and measures of statistical heterogeneity. If comparing groups, describe the direction of the effect. | n/a |
|  | 20c | Present results of all investigations of possible causes of heterogeneity among study results. | n/a |
|  | 20d | Present results of all sensitivity analyses conducted to assess the robustness of the synthesized results. | n/a |
| Reporting biases | 21 | Present assessments of risk of bias due to missing results (arising from reporting biases) for each synthesis assessed. | n/a |
| Certainty of evidence | 22 | Present assessments of certainty (or confidence) in the body of evidence for each outcome assessed. | n/a |
| **DISCUSSION** | | |  |
| Discussion | 23a | Provide a general interpretation of the results in the context of other evidence. | p18-20 |
|  | 23b | Discuss any limitations of the evidence included in the review. | p20 |
|  | 23c | Discuss any limitations of the review processes used. | p20 |
|  | 23d | Discuss implications of the results for practice, policy, and future research. | p19-20 |
| **OTHER INFORMATION** | | |  |
| Registration and protocol | 24a | Provide registration information for the review, including register name and registration number, or state that the review was not registered. | PROSPERO protocol ID, CRD42024535878. |
|  | 24b | Indicate where the review protocol can be accessed, or state that a protocol was not prepared. | n/a |
|  | 24c | Describe and explain any amendments to information provided at registration or in the protocol. | n/a |
| Support | 25 | Describe sources of financial or non-financial support for the review, and the role of the funders or sponsors in the review. | Title page |
| Competing interests | 26 | Declare any competing interests of review authors. | Title page |
| Availability of data, code and other materials | 27 | Report which of the following are publicly available and where they can be found: template data collection forms; data extracted from included studies; data used for all analyses; analytic code; any other materials used in the review. | n/a |

*From:*  Page MJ, McKenzie JE, Bossuyt PM, Boutron I, Hoffmann TC, Mulrow CD, et al. The PRISMA 2020 statement: an updated guideline for reporting systematic reviews. BMJ 2021;372:n71. doi: 10.1136/bmj.n71. This work is licensed under CC BY 4.0. To view a copy of this license, visit <https://creativecommons.org/licenses/by/4.0/>

**Appendix 2: Search Strategy**

**Ovid MEDLINE 17 January 2024**

| 1 | “Virtual ward*”.ab,kw,ti | 120 |
| --- | --- | --- |
| 2 | "hospital* at home".ab,kw,ti. | 810 |
| 3 | HaH.ab,kw,ti | 498 |
| 4 | “hospital* in the home”.ab,kw,ti | 266 |
| 5 | “Home hospital*”.ab,kw,ti | 635 |
| 6 | “Advance* care at home”.ab,kw,ti | 18 |
| 7 | “Virtual unit*”.ab,kw,ti | 12 |
| 8 | “Integrat* home care”.mp | 54 |
| 9 | “Virtual hospital*”.ab,kw,ti | 138 |
| 10 | (Virtual adj3 ward*).ab,kw,ti | 124 |
| 11 | 1 or 2 or 3 or 4 or 5 or 6 or 7 or 8 or 9 or 10 | 2290 |
| 12 | Elder* .ab,kw,ti | 316306 |
| 13 | "old* adult*".ab,kw,ti | 132321 |
| 14 | “old* person*”.ab,kw,ti | 15473 |
| 15 | “old* people*”.ab,kw,ti | 46324 |
| 16 | “old* age*”.ab,kw,ti | 102849 |
| 17 | senior*.ab,kw,ti | 53487 |
| 18 | "senior* citizen*".ab,kw,ti | 1774 |
| 19 | exp Geriatrics/ | 31691 |
| 20 | exp Geriatric Nursing | 13879 |
| 21 | Frail*.ab,kw,ti | 38136 |
| 22 | exp dementia/ | 210705 |
| 23 | Exp Frailty/ or exp frail elderly/ | 21482 |
| 24 | exp Aged/ | 3479198 |
| 25 | exp "aged, 80 and over"/ or centenarians/ or nonagenarians/ or octogenarians/ | 1018753 |
| 26 | 12 or 13 or 14 or 15 or 16 or 17 or 18 or 19 or 20 or 21 or 22 or 23 or 24 or 25 | 3835532 |
| 27 | 11 and 26 | 688 |
| 28 | Limit 27 to English language | **553** |

**Appendix 3: Data extraction topics**

| Study Title | Method of analysis |
| --- | --- |
| Author | Patient experience data |
| Publication year | Carer experience data |
| Source type | Staff experience data |
| Study design | Other key findings |
| Population: sample size, average age | Conclusions |
| Study’s definition of HaH model used | Implications to policy & practice |
| Health conditions | Strengths and limitations |

# **Appendix 4: Quality appraisal of literature assessed using Hawker Critical Appraisal Tool and AACODS for grey literature**

***Hawker Critical Appraisal tool results (excluding grey literature)***

| **First Author** | **Title**  **Abstra-ct** | **IntroAims** | **Meth-ods** | **Sam-pling** | **Anal-ysis** | **Ethics & Bias** | **Resu-lts** | **Generalis-ability** | **Useful-ness** | **Total** |
| --- | --- | --- | --- | --- | --- | --- | --- | --- | --- | --- |
| Chen [[26](#_ENREF_26)] | 4 | 4 | 4 | 3 | 4 | 4 | 4 | 3 | 4 | 34 |
| Dismore [[27](#_ENREF_27)] | 4 | 3 | 4 | 4 | 4 | 4 | 4 | 3 | 4 | 34 |
| Dowell [[28](#_ENREF_28)] | 2 | 4 | 3 | 2 | 3 | 2 | 3 | 2 | 2 | 23 |
| Gunnell [[29](#_ENREF_29)] | 3 | 2 | 3 | 3 | 3 | 2 | 4 | 2 | 4 | 26 |
| Jester [[30](#_ENREF_30)] | 2 | 4 | 4 | 4 | 3 | 2 | 3 | 3 | 2 | 27 |
| Karacaoglu [[31](#_ENREF_31)] | 4 | 4 | 4 | 3 | 4 | 2 | 4 | 2 | 4 | 31 |
| Kirkcaldy [[32](#_ENREF_32)] | 2 | 4 | 4 | 3 | 4 | 3 | 4 | 2 | 3 | 29 |
| Knowelden [[33](#_ENREF_33)] | 3 | 2 | 4 | 3 | 1 | 2 | 3 | 3 | 2 | 23 |
| Kotb [[34](#_ENREF_34)] | 4 | 4 | 4 | 4 | 4 | 2 | 4 | 4 | 4 | 34 |
| Makela [[35](#_ENREF_35)] | 4 | 4 | 4 | 4 | 4 | 3 | 4 | 3 | 4 | 39 |
| Ojoo [[36](#_ENREF_36)] | 4 | 2 | 4 | 4 | 3 | 3 | 3 | 3 | 3 | 29 |
| Saleh [[37](#_ENREF_37)] | 3 | 4 | 4 | 3 | 2 | 3 | 4 | 3 | 4 | 20 |
| Schofield [[39](#_ENREF_39)] | 4 | 4 | 4 | 3 | 4 | 2 | 4 | 3 | 4 | 32 |
| Schiff [[38](#_ENREF_38)] | 4 | 3 | 4 | 4 | 2 | 2 | 3 | 4 | 3 | 29 |
| Shepperd [[41](#_ENREF_41)] | 4 | 3 | 4 | 4 | 4 | 3 | 3 | 3 | 4 | 32 |
| Shepperd [[40](#_ENREF_40)] | 4 | 4 | 4 | 4 | 4 | 4 | 4 | 3 | 4 | 35 |
| Vindrola-Padrosa [[42](#_ENREF_42)] | 4 | 4 | 4 | 3 | 4 | 4 | 4 | 4 | 4 | 35 |
| Wilson [[43](#_ENREF_43)] | 4 | 3 | 4 | 3 | 2 | 3 | 4 | 2 | 3 | 28 |

***Quality appraisal of grey literature using AACODS***

| **Health Foundation [**[**44**](#_ENREF_44)**]** | **Health Innovation Network [**[**45**](#_ENREF_45)**]** |
| --- | --- |
| Reputable authority- an independent charitable organisation in UK [[78](#_ENREF_78)]. | Reputable authority- a body that connects NHS with academic organisations, local authorities, charities, and industry [[47](#_ENREF_47)]. |
| Meaningful, balanced insight into views of the public and NHS staff on virtual wards that are not widely covered in formal literature. Clear aims in stated methodology | Adds to the evidence base of patient and staff perspectives (carer perspectives were limited in this report). Clear aims in stated methodology. Innovative practice recommendations. |
| Generalisable to UK population. Sample was representative of age, gender, ethnicity, region, and socioeconomic group. A full population breakdown was not provided so limits extent of conclusions drawn. | Croydon’s diverse patient population adds to generalisability as the sample was relatively representative. A full demographic breakdown would have been valuable to fully assess this. |
| No conflict of interest mentioned. Survey commissioned with Censuswide with no detail on its affiliation with The Health Foundation. | Conflict of interest may arise as it is an internal evaluation so reporting may be in favour of these organisations as it assesses HaHs run by them. |
| No limitations stated. | Methodological bias due to rapid evaluation design. |

**Appendix 5: Summary table of quotes for each theme**

| **Theme** | **Quote** |
| --- | --- |
| Familiarity of home versus a stark hospital environment | “*Her recovery time was much quicker so it was easier for me because she was helping herself do you know what I mean*” (Carer to patient in a COPD HaH) [[27](#_ENREF_27)].  *“You’re in your own surroundings which helps you get better quicker” (*HaH patient)[[43](#_ENREF_43)]  “*People recover better in their own home and there was one lady and I quote her often to the staff there’s no bed like your own bed… sitting in a hospital bed when you’ve got poorly people around you if you have that support to be able to recuperate at home*” (Manager of COPD HaH) [[27](#_ENREF_27)].  “*It’s easier for my family to visit... getting cars parked... when they have been to work all day by the time they get the hospital it’s time to come home*” (Patient in COPD HaH)[[27](#_ENREF_27)]  “*Some people maybe prefer to be in a cocoon of a hospital environment, they maybe worry if something goes wrong someone’s seconds away from them*” (Patient) [[27](#_ENREF_27)]  *Having his family around him was the most important thing for him, and Hospital at Home allowed that to happen”* (Relative to patient in COVID-19 HaH) [[38](#_ENREF_38)].  *"I’d prefer to be looked after at home because I feel more contented…I feel my own wee home is familiar to me, I know everything and I’m contented in it so I feel more happier if I’m in my home"* (HaH patient) *[*[*39*](#_ENREF_39)*]*  “…*had my wife been in hospital, I’d still be doing the jobs at home and looking after the house and I’d still have to go to the hospital to visit her and the time I’ve spent in the hospital visiting her, I could rest at home.’* (Family carer to wife admitted to HaH) [[43](#_ENREF_43)]  *‘I didn’t like it in hospital because I was alone in the room and had nobody to talk to. They would come into my room, go to the locker, take out the tablets and plonk them down. They never said anything, not even “Good morning” ...’* (Hospital patient)[[43](#_ENREF_43)]  “*It’s better my husband being at home and getting the treatment at home instead of like going back and forward to the hospitals and especially with being a carer looking after two children as well you know it’s a lot better”* (Patient in COPD HaH) [[43](#_ENREF_43)].  “*If there is something wrong with his heart he should be in hospital, where all the necessary equipment is…”* (HaH carer) [[43](#_ENREF_43)]. |
| Person-centred care | “*…you get closer to the patients when you’re in the home because... you spend more time with them, so you get to know them better as they tend to open up to you … which I don’t think if you’re on a ward, that they would…they’d be like, oh, they’re too busy…but if you’re there(at patient’s home) …they open up to you”* (HaH manager) [[26](#_ENREF_26)].  “*I’ll be perfectly honest the times I’ve been in the hospital the nurses there been too busy… you don’t get a one to one erm but in your home you do get one to one*.” (Patient) [[27](#_ENREF_27)].  *“I think you got more attention [from Hospital at Home] ... it seems as if you are the only one, but you’re not ... they’ve got a lot of patients to see but I’m more than satisfied with all of them ... ”* (HaH patient) [[43](#_ENREF_43)]  *“They were marvellous, the home care nurses, they couldn’t have done more for him ... and they looked after me.”* (HaH family carer) [[43](#_ENREF_43)] |
| Enabling shared decision making | *“I think lots of our patients and carers have definitely felt that they’ve been more involved than they might have been if they’d come into hospital. Definitely having them at home, I think they’ve just found more reassuring, because they can be more involved in all of that decision making and caring, which might otherwise have been taken away from them...”* (Consultant geriatrician clinical lead for HaH) [[26](#_ENREF_26)]  *“They all like continuity, they like the same person going in...they look forward to you coming”* (Healthcare support worker in HaH) [[31](#_ENREF_31)]  *“We educate the patient so that they can continue that throughout the day... they’re able to manage their oxygen saturations and read some numbers off. And they actually then develop a certain sense of control and autonomy in their illness rather than being a kind of very passive participant in their illness in that...in the hospital bed.”* (Doctor running Acute Medical Unit HaH*) [*[*26*](#_ENREF_26)*]*  “*Think the only concern I would have is that hospital at home is only as good as the nurses you’ve got on”* (HaH patient) [[27](#_ENREF_27)]  *“It gives us a chance to be more involved in treatment and understanding the problem and allowing us some control which I often think is missing”* (HaH patient onboard an atrial fibrillation HaH) [[34](#_ENREF_34)] |
| Challenges with staff and  information accessibility | “*All (HAH) did was wrote down on a piece of paper, took it away, we never see it no more. . . The doctor’s been given a copy, but surely we should have a copy so we’ve got an account of it”* (Son of patient in HaH) [[35](#_ENREF_35)].  *“It will be very slow until it feeds into the GP practices. We could take a lot of load from them if they meet us half way”* (Healthcare support worker in HaH) [[31](#_ENREF_31)] |
| Safety of HAH | *“I had confidence in the team that came out... they all seemed to me to be very well trained and put me at ease:* (Patient in COPD HaH) [[27](#_ENREF_27)]  “*it’s like sleeping with one eye open…”* (Son to a patient onboard an admission avoidance HaH) [[35](#_ENREF_35)]  *“…quite a lot of work isn’t it, running up and down for me... I’m worried if she falls… I don’t think I could lift her”* (Husband to patient onboard an admission avoidance HaH) [[35](#_ENREF_35)]  *“You felt calm, you knew somebody was coming, at ease. You’re not waiting for a nurse to come and help you to do things like you are in hospital.*  *”* (HaH patient) [[43](#_ENREF_43)].  *“I’d got the knowledge that if I did need any help I could get it. They left me a telephone number, they really stressed that when they left, if you want any help don’t forget to ring us ... (I was) confident they would know if I was very ill and that they would move me to hospital, that they would get the doctor.”* (HaH patient) [[43](#_ENREF_43)]  “*The only thing I would fault was the night if you were really needing it.. not to be there all the time but to pop in*” (HaH patient) [[43](#_ENREF_43)]  “*every time they (healthcare professionals) went I was upset, because I was so alone. I was rigid with nerves*” (HaH patient) [[43](#_ENREF_43)]. |
| Managing technology at home | *“I am a technophobe and I found it easy”* (Patient onboard an atrial fibrillation HaH) [[34](#_ENREF_34)]  “*I found it difficult at first but was able to get support from staff once I got home*.” (Patient onboard an atrial fibrillation HaH) [[34](#_ENREF_34)]  *“I feel like elderly patients might get confused with the technology*.” (Patient feedback from an atrial fibrillation HaH) [[34](#_ENREF_34)]  *“Equipment was simple to use, and the feedback was helpful and reassuring”(HaH patient) [*[*45*](#_ENREF_45)*]*  *“It’s so easy am not computer literate so if I can do it anyone can and the nurse that showed me was great”* (HaH patient) [[45](#_ENREF_45)] |
